# Exploring the Role of Insulin Resistance in Fueling Stroke Vulnerability and Worsening Post-stroke Prognosis: A Narrative Review of Current Literature

**DOI:** 10.7759/cureus.48034

**Published:** 2023-10-31

**Authors:** Mihir H Sojitra, Vasudha S Garg, Karan Shah, Saumya Joshi, Harsh Vadnagara, Siddharth Kamal Gandhi, Priyansh Patel

**Affiliations:** 1 Department of Neurology, Civil Hospital Ahmedabad, Ahmedabad, IND; 2 Department of Internal Medicine, Civil Hospital Ahmedabad, Ahmedabad, IND; 3 Department of Internal Medicine, Shri M. P. Shah Government Medical College, Jamnagar, IND; 4 Department of Internal Medicine, Medical College Baroda, Vadodara, IND

**Keywords:** end neurological decline, cerebro-vascular accident (stroke), stroke, insulin resistance, post stroke prognosis

## Abstract

Stroke remains one of the world's greatest causes of disability and death. Insulin resistance (IR) impairs insulin's beneficial effects on the brain and can change the course of illness in post-stroke patients. This review aims to find sufficient evidence to support the causal association of IR in ischemic stroke and with post-stroke prognosis (PSP). The review will also list probable mechanisms to better understand how IR affects stroke pathology. Various articles from PubMed Central, MEDLINE, and PubMed databases were reviewed, and then after careful consideration, 17 articles were selected. The studies, using various genetic and metabolic markers, have linked IR to increased incidence of ischemic stroke. Among the various types of strokes investigated from this standpoint, silent lacunar infarct stands out as a widely researched subtype. Even though the exact pathogenesis is still unclear, current evidence shows an interplay of atherosclerosis, embolism, and platelet dysfunction. The development of early neurological decline (END) in post-stroke patients has been used to link IR to poor PSP. It is also acknowledged to have contributed in some way to poor three-month outcomes. Modifying inflammatory pathways and developing glucotoxicity are some of the pathways by which IR affects PSP. After reviewing the studies, significant evidence was found to support the role of IR in causing ischemic stroke as well as in poor PSP. Additional investigation is required to assess its influence on three-month prognosis and its significance in various stroke subcategories.

## Introduction and background

Despite significant advances in clinical interventions aimed at reducing stroke risk factors such as hypertension, smoking, and diabetes, stroke incidence and patient prognosis have not changed significantly over the last few decades [1]. Stroke remains the world's second-greatest cause of disability and death [1]. Each year, more than 14 million people around the world experience an ischemic stroke [2]. An estimated 6.6 million Americans over the age of 20 experience a stroke, which occurs every 40 seconds on average and kills a person every four minutes [3]. Recent advances and expanding research are being conducted to determine the mechanism of insulin resistance (IR) in multiple brain illnesses. Because glucose is the primary energy source in the brain, abnormal glucose metabolism in IR becomes an essential risk factor in brain illnesses, such as ischemic stroke [1]. IR weakens the beneficial function of insulin. Insulin safeguards the growth of brain tissue by shielding it against ischemia, oxidative stress, and damage caused by apoptosis. Moreover, insulin plays a role in regulating cholesterol metabolism in neurons and astrocytes, thereby enhancing the protection of brain tissue [1]. As a potential causal link, it is critical to comprehend the role of IR in ischemic stroke. This topic has been the subject of extensive research. However, there isn't sufficient knowledge summarizing the various studies done to prove this correlation. The exact mechanism by which IR causes its effect is also unclear. This review attempts to shed light on the current research to find sufficient evidence to support the causal association of IR in ischemic stroke and with post-stroke prognosis (PSP). Apart from this, a few probable mechanisms have also been listed to provide a better understanding of how IR affects stroke pathologically.

Methodology

In collaboration with all the authors, PubMed Central, MEDLINE, and PubMed databases were searched. Firstly, a regular keyword search for "ischemic stroke and insulin resistance" showed 247 articles. Thus, another strategy was included based on medical subject headings (MeSH) vocabulary: ("Ischemic Stroke" [Majr]) AND ("Insulin Resistance" [Majr]) for more precise results. No time limits were set. The initial search criteria showed 30 articles. Then, applying a filter of free full text, nine articles were then discarded. Abstracts of all the remaining articles were studied for duplicates and relevance to the study. After careful consideration and discussion, 17 articles were selected.

## Review

IR and brain: normal insulin physiology

The control of energy balance and peripheral glucose metabolism is governed by the insulin hormone, which is secreted by pancreatic beta cells [1]. Additionally, the CNS plays a role in regulating this process, and the insulin hormone can pass through the blood-brain barrier and access the brain tissue [1]. In conjunction with plasma membrane insulin receptors, insulin exerts its effects. Insulin promotes neuronal survival, regulates the release of catecholamines, and stimulates neural growth. When tissues do not respond normally to insulin stimulation, such as brain tissue, IR occurs [1]. This IR has been extensively investigated as a risk factor for a variety of cardiovascular diseases, but we will focus on its function in the development of ischemic stroke in this study.

Association between IR and ischemic stroke

Insulin is an endocrine hormone secreted by beta cells of the pancreas, primarily secreted as a hormone to lower blood sugar levels. The intricate mechanisms governing energy balance involve insulin signaling originating from the CNS [1]. Being a regulator of glucose metabolism, the energy bank of the brain, insulin's role in normal brain activity is extremely vital. Hence, when the functioning of insulin gets altered in IR, the chances of organic diseases developing in the brain increase [1]. One such important disease is ischemic stroke. There have been several studies linking IR to ischemic stroke. Since IR as such cannot be quantified, its presence has been defined by genetic as well as metabolic markers which were then used to develop the same causal relationship with ischemic stroke.

Two distinct Mendelian studies were conducted to investigate the potential links between IR and ischemic stroke, encompassing various subtypes of the condition. To implement these studies, researchers chose 53 single nucleotide polymorphisms linked to the IR phenotype identified in various genome-wide association studies. These selected polymorphisms served as instrumental variables in the Mendelian randomization analysis, which aimed to determine any associations between IR and ischemic stroke and its subtypes [4,5]. A specific study demonstrated a connection between heightened IR, measured through fasting blood glucose levels, and an increased likelihood of developing ischemic stroke, including both large artery stroke and small vessel stroke. After analyzing further, they found that IR mediated by altered fat distribution was associated with a higher risk of small vessel stroke, whereas obesity did not show much significance with large vessel stroke and cardioembolic stroke [4]. There was another study that sought to explore the associations between IR and cardiovascular diseases. It was discovered that one standard deviation (SD) increase in IR phenotypes was significantly linked to a substantial rise in the risk of ischemic stroke, particularly the small artery occlusion subtype of stroke [5]. However, unlike previous studies, they could not find any significant association for larger artery atherosclerotic and cardioembolic subtypes of stroke [5].

The association was further supported indirectly by using surrogate markers predicting IR like triglyceride index (TgY), homeostasis model assessment-estimated IR (HOMA-IR), and IR score. Upon analysis, TgY level with subsequent stroke in a community-based prospective cohort which followed participants for 11.02 years showed that higher TgY index at baseline was associated with increased risk of development stroke and ischemic stroke in the future [6]. Another index is the HOMA-IR index, widely used in clinical and epidemiological research. This index was used in a cross-sectional Korean study that showed that IR was independently associated with the presence and severity of silent lacunar infarct (SLI), a subtype of stroke leaving room for other studies to describe causal relationship in the future [7]. The relationship of IR with stroke was further supported by a Japanese prospective cohort study that showed that reduced insulin sensitivity and muscle strength independently increased the risk of SLI in elderly patients [8]. The study showed a 4.33-fold increase in stroke among candidates with lower insulin sensitivity (measured by the PREDIM index) and muscle weakness versus those with higher levels of both parameters [8]. Another study demonstrated that midlife central obesity and IR are related to incident lacuna, especially larger lesions like micro atheromatous disease. The combined effects of these factors were better captured using an IR score. Participants with higher IR scores were more likely to experience larger effects with both larger and smaller lacunas [9].

Since the TgY index did not take into account the effects of diet and cholesterol on cardiovascular disease, a novel marker called metabolic score for IR (METS-IR) was created. METS-IR combined fasting plasma glucose, TgY, high-density lipoprotein cholesterol (HDL-C), and BMI, thus overcoming the limitations of the TgY index [10]. This highly predictive index was used as a predictor of IR in a study on Chinese hypertensive patients to show its association with stroke and its subtypes. During a median follow-up of 4.8 years, this observational retrospective cohort study found an increased risk of stroke and stroke subtypes among participants with higher METS-IR at baseline [10].

Pathogenesis of IR in ischemic stroke

**Figure 1:**
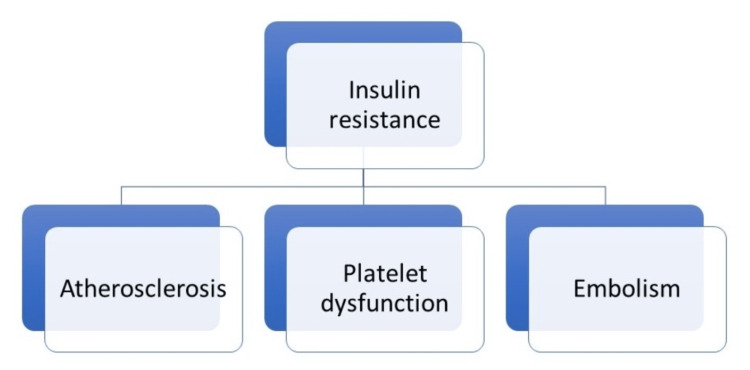
Virchow's triad promoted by IR Image credit: Mihir Sojitra and Priyansh Patel

Among the various literature published on the role of IR in ischemic stroke, the most relevant mechanism was that IR caused atherosclerosis, platelet dysfunction, and a hypercoagulable state which is also known as Virchow’s triad [1], as shown in Figure 1.

Atherosclerosis

**Figure 2:**
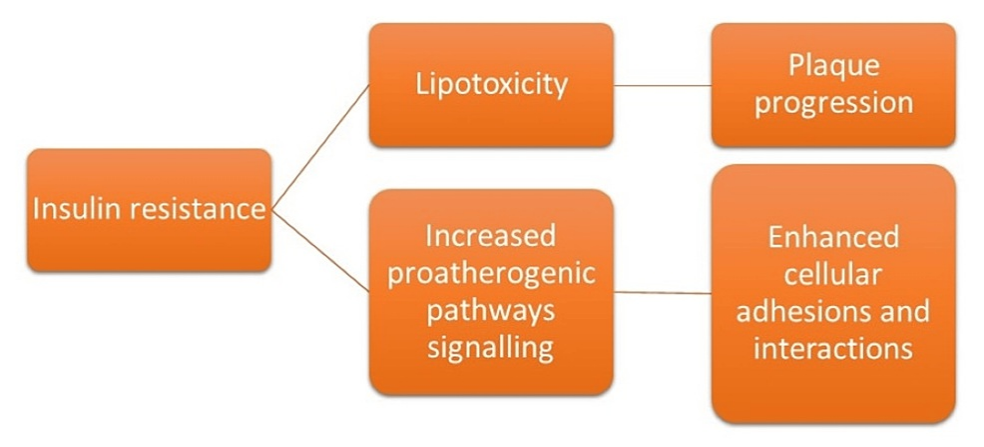
IR and its pathogenesis Image Credit: Mihir Sojitra and Priyansh Patel

Atherosclerosis is characterized by the gradual narrowing of arteries due to the deposition of fatty material on their inner walls. This has a complex pathogenesis in which various intermediates play a role. The most important initiating factor of atherosclerosis is endothelial dysfunction [11]. IR leads to decreased insulin effects leading to lipolysis and thus enhanced low-density lipoprotein (LDL) products causing lipotoxicity. This, along with lipotoxicity and various proinflammatory cytokines, plays an important role in causing endothelial dysfunction [7]. Apart from initiating plaque formation, it also played a role in plaque progression as showed by previous studies in which TgY was an independent marker for the progression of plaque [6]. Secondly, there have been various proatherogenic pathways that portray a role in atherosclerosis. One such inflammatory pathway is the mitogen-activated protein kinase (MAPK) signaling pathway. IR has been shown to enhance this MAPK signaling pathway, thereby enhancing cell adhesion and interaction aids in atherosclerosis [7]. The mechanism by which IR causes plaque formation and cellular interactions is shown in Figure 2.

Platelet Dysfunction

Platelets are known for their role in coagulation, and when they malfunction, coagulation can occur in the wrong places. Normally, insulin reduces platelet aggregation by transferring magnesium intracellularly. Insulin also improves the platelet sensitivity of platelet inhibitors such as prostacyclin (PGI2) and enhances their synthesis [1]. However, in IR, this inhibitory effect is removed. Hence, there is hyperactivation of platelets by changes in multiple processes in platelet activation such as adhesion, activation, and aggregation. This leads to micro- and macrovascular events [1,7]. This causes future vascular stenosis, predisposing the patient to ischemic stroke. A previous analysis of several genetic polymorphisms of thromboxane A2 in normal and metabolic syndrome patients revealed that a specific polymorphism (NC_000007.14:g.139985896C>T) was related to ischemic stroke [12].

Hypercoagulable State

The role of hypercoagulable state in ischemic stroke is well understood. Because blood clots are the most prevalent type of emboli, the majority of research has been able to demonstrate a link between stroke and emboli by eliciting the pathology of thrombosis [1]. The probable link is that IR, a hypercoagulable and pro-atherogenic state, might lead to intravascular thrombus. The dislodgement of this thrombus would cause an embolic event in major vessels, resulting in a stroke [1]. The relevance of IR as an independent risk factor for early neurologic deterioration (END) in patients with non-diabetic acute ischemic stroke was also supported by another study [3]. Nevertheless, the aforementioned study also observed a lack of substantial evidence to corroborate the utilization of IR as an independent predictor of an adverse prognosis at the three-month mark [3]. In the same analysis, the prior negative findings were confirmed by a Japanese study that revealed no statistically significant evidence linking HOMA-IR to stroke recurrence or three-month death in individuals with acute ischemic attack [3].

Association between IR and post-stroke patient

To understand the role of IR in a patient’s PSP, several studies were done, all of which showed that it was independently associated with poor outcomes in ischemic stroke patients.

Outcomes Associated With Ischemic Stroke

END, stroke recurrence, post-stroke depression, and all-cause death are short-term outcomes associated with ischemic stroke [3]. In the trials, END is defined as cognitive decline or recurrence during the first 72 hours following an ischemic stroke. Studies have found that several factors can help predict the development of END, including the initial severity of the stroke, the origin of the stroke, metabolic factors, hemodynamics, radiographic data, extensive lesions, and vascular occlusions. These factors influence END risk and increase the overall risk of stroke-related complications [13]. The initial severity of stroke can be scaled by a physician using the National Institutes of Health Stroke Sc (NIHSS) score on admission and at discharge. The deterioration can be described by more than or equal to a two-point increase in NIHSS score at discharge compared to admission [2,14]. Most studies have tried to find a relation with poor functional outcomes after three months from stroke onset. To predict functional outcomes, stroke neurologists and trained research nurses were appointed who assessed the condition based on a modified Rankin scale (mRS). Poor functional outcome was defined as a mRS score of three to six [2,14].

Association Between IR and END and Poor Outcomes of Stroke

A prospective study aimed at understanding the effects of IR on recovery in non-diabetic post-ischemic stroke patients showed that when the clinical course was assessed by the presence of END, outpatient visits, or centralized telephone services to conduct the three-month follow-ups, it showed that IR, calculated in terms of HOMA-IR scores, was associated with poor functional outcome in non-diabetic ischemic stroke patients [14]. In addition, another study revealed that patients with diabetes mellitus (DM) and metabolic syndrome experienced greater rates of lacunar stork recurrence while receiving medical care. Since IR is significant in both metabolic syndrome and DM, this information indirectly supports the idea that IR contributes to stroke patients' poor clinical outcomes. However, because these patients patients' have multiple risk factors, more research on the independent function of IR is needed [15]. As a result, even while IR can be an independent risk factor for END in post-stroke patients, its significance in poor three-month outcomes is yet unclear. Guidelines and a strategy for comprehensive reviews and meta-analyses have been established to encourage studies to identify more compelling evidence of a link between IR and PSP in patients without diabetes [16].

Role of IR in Pathogenesis of Poor Clinical Outcome in Stroke Patients

**Figure 3:**
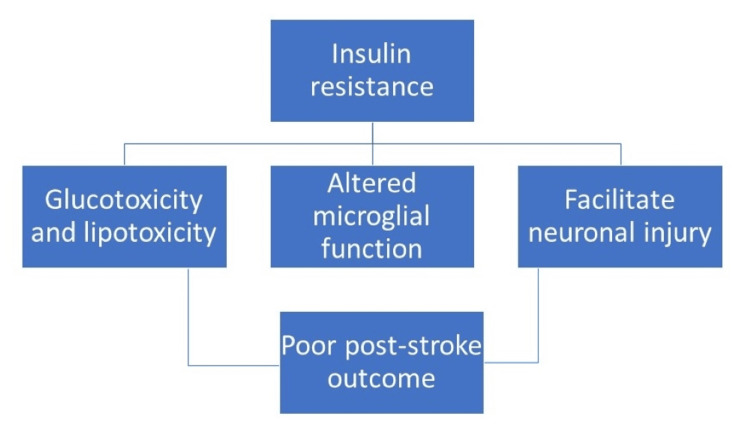
Association of IR in PSP Image credit: Mihir Sojitra and Priyansh Patel

Inflammation and oxidative stress are two major ways that IR has influenced the course of a stroke. At the molecular level, these intricate processes have been studied. Figure 3 describes the role of IR in PSP.

Glucotoxicity and Lipotoxicity Leading to Reactive Oxygen Species (ROS) Stress

IR is an altered glucose metabolism with decreased expression of glucose transporters resulting in decreased glucose uptake by skeletal muscle, adipose tissue, liver, and other organs [1,3]. IR also results in uninhibited gluconeogenesis and decreased glycogen synthesis which ultimately results in excess levels of glucose in peripheral blood. This hyperglycemia is a known culprit in causing oxidative stress [17]. The mechanisms could be increased production of amadori, an intermediate in the production of advanced glycation end products, which is known to produce ROS. Another mechanism is increasing the production of the physiologic ROS produced in the electron transport chain in mitochondria [17]. As discussed, IR can cause hyperglycemia, but it was also found in studies that patients with chronically elevated glucose had decreased insulin sensitivity. Hence, hyperglycemia and IR have a bidirectional relationship [3]. IR has decreased inhibitory effects on lipolysis. This leads to elevated levels of LDL, free fatty acids (FFA), TgY, and decreased HDL levels. Increased FFA levels activate the NADPH-ROS pathway, increasing the production of ROS [3]. Adipocytes sense this oxidative stress and activate the proinflammatory cytokine nuclear factor-κB (NF-κB) pathway and proinflammatory proinflammatory cytokines like NLRP3, tumor necrosis factor (TNFproinflammatoryn (IL)-1β, and IL-6. All these alterations lead to endothelial dysfunction and increase the risk of future atherosclerosis and stroke [3].

Alteration of Microglial Function

An essential part of the immune response against the ischemic insult is played by microglia, a distinct type of immune cell. They can remove dead tissue thanks to their phagocytic and antigen-presenting capacities [1]. They also contribute to the development of the inflammatory process by releasing a variety of proinflammatory cytokines, including TNF-a and IL-1b, which aid in the attraction of additional immune cells [1]. They can be classified as either classically activated (proinflammatory, M1 phenotype) or activated (proinflammatory, M1 phenotype) phenotypes based on polarisation patterns [1]. During an ischemic insult, the M1 type predominates. Since microglia are the main mediators of inflammation, it is crucial to realize how IR impacts their function to comprehend how it influences the inflammatory process following an ischemic stroke [1]. By preventing macrophage polarization to the M2 phenotype and pushing them into the M1 type, IR increases cellular damage by preventing glucose oxidative metabolism. By releasing the chemokine, monocyte chemo-attractant protein 1 (MCP1), IR causes a local buildup of M1-type microglia [1]. The likely explanation for inflammation induced by IR, about alteration in the metabolic profile of macrophage, is that IR restricts the transport of glucose into neurons, hindering the process of oxidative phosphorylation [1].

IR and central neurons

Understanding how insulin affects central neurons could help us better understand how insulin influences stroke prognosis in addition to its many peripheral and metabolic effects. Patients with ischemic stroke may experience decreasing neurological function and a worse functional result at three months due to neuronal damage caused by IR [1]. Due to decreased sensitivity, there is compensatory secretion of insulin by the pancreas. This hyperinsulinemia leads to the development of IR in central neurons. One notable impact is the diminished activity of the phosphoinositide 3 kinase (PI3K/AKT) signaling pathway. Normally, the AKT prevents cell death by phosphorylating pro-apoptotic signaling molecules, including forkhead transcription factor (FKHR), GSK-3β, and Bad. The decreased expression of AKT leads to reduced neuronal survival [2]. The oxygen-glucose deprivation in ischemic IR tissue also suppresses the AKT pathway, which results in lower mTORC1 activity (a classical pathway that prevents the activation of autophagy). The glucose concentration in neurons drops due to the reduced activity of the GLUT-4 membrane glucose transporter, which causes neurons to apoptose [1]. Learning and memory are notably influenced by synaptic plasticity, where alterations in the efficacy of neural connections are contingent on neural activity. Knowing that insulin is necessary for the function of neuronal synapses, IR also affects neuronal function and synaptic plasticity [1]. The loss of insulin’s neuroprotective properties in IR facilitates ischemic damage [3].

Limitations

Several constraints were observed in the study. The literature covered was only taken from three databases: PubMed Central, MEDLINE, and PubMed databases. Due to full-text filters, the articles that required subscriptions were filtered as well. Apart from these, articles were removed due to the language barrier as only articles in English were chosen. With a greater amount of studies from the southwestern hemisphere, it is difficult to generalize. Consequently, further larger studies with more homogenous populations are required to make a solid connection between IR and PSP.

## Conclusions

IR is well-known for its role as a risk factor for stroke and also influences the prognosis following a stroke. Based on several investigations, it might be suggested that IR contributed to the development of atherosclerosis which had a metabolic role in the pathogenesis of ischemic stroke. The role of IR in lacunar stroke among the lacunar infarct, a subtype of stroke, is sufficiently supported by the available data. IR is a key factor in the emergence of END, but further research is required to determine how it affects prognosis at three months. One of the key ways that IR influences the PSP is by altering the inflammatory pathways and insulin sensitivity of central neurons. However, more investigation is necessary regarding its separate pathogenesis in different subtypes of stroke.
